# Lutein and Zeaxanthin—Food Sources, Bioavailability and Dietary Variety in Age-Related Macular Degeneration Protection

**DOI:** 10.3390/nu9020120

**Published:** 2017-02-09

**Authors:** Bronwyn Eisenhauer, Sharon Natoli, Gerald Liew, Victoria M. Flood

**Affiliations:** 1 Food and Nutrition Australia, Sydney NSW 2000, Australia; beisenhauer@foodnut.com.au (B.E.); snatoli@foodnut.com.au (S.N.); 2 Centre for Vision Research, Department of Ophthalmology, Westmead Millennium Institute, The University of Sydney, Sydney NSW 2145, Australia; gerald.liew@sydney.edu.au; 3 Faculty of Health Science, The University of Sydney, Sydney NSW 2141, Australia; 4 Westmead Hospital, Western Sydney Local Health District, Westmead, Sydney NSW 2145, Australia

**Keywords:** lutein, zeaxanthin, xanthophylls, carotenoids, age-related macular degeneration, bioavailability

## Abstract

Lutein and zeaxanthin (L/Z) are the predominant carotenoids which accumulate in the retina of the eye. The impact of L/Z intake on the risk and progression of age-related macular degeneration (AMD), a leading cause of blindness in the developed world, has been investigated in cohort studies and clinical trials. The aims of this review were to critically examine the literature and evaluate the current evidence relating to L/Z intake and AMD, and describe important food sources and factors that increase the bioavailability of L/Z, to inform dietary models. Cohort studies generally assessed L/Z from dietary sources, while clinical trials focused on providing L/Z as a supplement. Important considerations to take into account in relation to dietary L/Z include: nutrient-rich sources of L/Z, cooking methods, diet variety and the use of healthy fats. Dietary models include examples of how suggested effective levels of L/Z can be achieved through diet alone, with values of 5 mg and 10 mg per day described. These diet models depict a variety of food sources, not only from dark green leafy vegetables, but also include pistachio nuts and other highly bioavailable sources of L/Z such as eggs. This review and the diet models outlined provide information about the importance of diet variety among people at high risk of AMD or with early signs and symptoms of AMD.

## 1. Introduction

Lutein and zeaxanthin (L/Z) are two fat-soluble antioxidants belonging to the class of carotenoids called xanthophylls. Along with their conversion isomer meso-zeaxanthin, they are the major constituents of macular pigment, a compound concentrated in the macula region of the retina that is responsible for fine-feature vision. Given their accumulation in the retina, the role L/Z play in eye health has been investigated with a particular focus on how consumption of these carotenoids may prevent and/or slow the progression of age-related macular degeneration (AMD), the leading cause of blindness in older adults [1]. Recently published prevalence rates for early AMD in Irish adults over 50 years and New Zealand adults 45−85 years are 7.2% [2] and 10.3% [3], respectively. Furthermore, as the leading cause of severe vision impairment in Australians over 40 years of age, AMD continues to be an important target for the development of effective preventative therapies to ease the medical and economic burden of this disease in the developed world [4]. US research has forecast that the number of patients with AMD is likely to double between 2010 to 2050 and this disease is becoming a crucial public health issue [5]. Recent review articles have detailed the evidence linking L/Z and AMD with an emphasis on L/Z supplements, the transport and storage of L/Z in the macula as well as the potential mechanisms of action they have in AMD [6,7,8,9]. These papers provide limited details regarding the potential role of dietary sources of L/Z in AMD. The aim of this paper is to summarise the current available evidence regarding L/Z’s role in AMD and, specifically, to hypothesise and model a likely suitable amount of lutein and zeaxanthin to obtain from dietary sources that may reduce the risk of AMD (progression and prevention), taking into account common food sources, such as vegetables and eggs, and factors affecting their absorption and bioavailability in the body.

## 2. Dietary Carotenoids 

Around 700 carotenoids have been characterised in nature; approximately 20−30 have been identified in human blood and tissues, but only lutein and zeaxanthin are found in the eye [9,10]. L/Z (with the isomer meso-zeaxanthin) accumulate in the retina of the eye and form the retinal macular pigment (MP) [11]. MP has several functions in improving visual performance and protecting against the damaging effects of light, and MP levels are used as a proxy for macular health—specifically to predict the likelihood of developing AMD [11]. In the retina, these three compounds (lutein, zeaxanthin and meso-zeaxanthin) exhibit regional dominance with meso-zeaxanthin being the dominant carotenoid at the epicentre, zeaxanthin at the mid-periphery and lutein at the periphery of the macula [12].

## 3. Age-Related Macular Degeneration (AMD)

AMD is one of the leading causes of blindness in older adults in the developed world [13]. AMD increases with advancing age and appears to be more likely in men than women [14], although this differs between countries [15]. Table 1 details a clinical classification scale for AMD. Early AMD is generally asymptomatic while late AMD leads to loss of central vision and potential blindness. Images of intermediate and neovascular late AMD are provided in supplementary material as Figures S1 and S2.

Synergistic associations between diet, lifestyle and genes and risk of AMD have been shown, with unhealthy lifestyles associated with an increased AMD risk regardless of AMD risk genotype [17]. A number of dietary measures have been associated with slowing the progression of AMD. These include a diet rich in omega-3 fatty acids [18], a diet with a lower dietary glycemic index [19,20] and intake of nutrients including vitamins C and E, beta-carotene, zinc, selenium, B vitamins, folate and vitamin B12 and lutein and zeaxanthin [21,22,23,24].

Their unique presence in the centre of the macula implies an important role for L/Z in visual performance [7]. Antioxidant and blue light-filtering properties of L/Z for short wavelengths are hypothesised to protect the eye against AMD [9]. The filtration of blue light reduces chromatic aberration which can enhance visual acuity and sensitivity [25]. The following review focuses on L/Z and includes a summary of the observational evidence obtained from cohort and case-control studies as well as data obtained from randomised controlled trials which have investigated the relationship between dietary and supplemental L/Z and AMD development and/or progression.

## 4. Lutein and Zeaxanthin and AMD—Epidemiological and Intervention Studies

Evidence of an association between the consumption of fruits and vegetables and the risk of AMD was reported in 1988 [26] when data obtained from the first US National Health and Nutrition Examination Survey was published. Following this, a number of cohort and case-control studies have examined associations between dietary intake of L/Z and risk of AMD [21,27,28,29,30,31]. These studies are summarised in Table 2.

A 2012 systematic review and meta-analysis of six longitudinal cohort studies observed that the protective effects of high intakes of dietary L/Z may be confined to late AMD [32]. Researchers calculated a pooled relative risk (RR) for early AMD, comparing the highest with the lowest category of L/Z intake, 0.96 (95% confidence interval (CI) 0.78, 1.17), whereas dietary intake of these carotenoids was significantly related with a reduction in risk of late AMD (RR 0.74; 95% CI 0.57, 0.97). Furthermore, the study found a 32% risk reduction of neovascular AMD (RR 0.68; 95% CI 0.51, 0.92) [32] among those people who consumed the highest category of L/Z compared to those who consumed the lowest. 

Variation is seen in the results from cohort and case-control studies available to date. Overall, the evidence suggests a potential protective effect of L/Z consumption particularly in relation to younger cohorts, late AMD and those at high genetic risk of AMD.

Further evidence that L/Z intake may provide protection against the development of AMD comes from the Age-Related Eye Disease Study 2 (AREDS2)—a major clinical trial conducted in the US. In the original Age-Related Eye Disease Study (AREDS1), researchers found that supplementation with vitamins C (500 mg) and E (400 IU), β-carotene (15 mg), zinc (80 mg) and copper (2 mg) at levels well above the recommended daily allowances reduced the risk of progression to a more advanced AMD by about 25% [23]. Later, AREDS2 incorporated lutein (10 mg) and zeaxanthin (2 mg) into the formulation being used in the study among people with early AMD [38]. In secondary analysis, L/Z supplements on top of the AREDS supplement lowered the progression to advanced AMD, though this only occurred in persons with low dietary L/Z [38]. A reduction in risk was seen for individuals in quintile 1 who had a median intake of 696 µg L/Z per 1000 cal per day (interquartile range 552−823 µg/1000 cal per day) with a hazard ratio (HR) of 0.74 (95% CI, 0.59−0.94) when compared with no L/Z intake. For those with a median intake of 1134 μg per 1000 cal per day (interquartile range 1030−1244 µg/1000 cal per day) the HR was 0.94 (95% CI, 0.74−1.21) showing that the supplement had no additional impact of progression of the disease when the background diet was sufficient in L/Z. This implies that dietary intakes of at least 2268 µg of L/Z (with a range of 2060–2488 µg, assuming an average daily energy intake of 2000 calories) may provide some level of protection against late AMD, but this needs to be confirmed in other dietary trials of L/Z among people with AMD, or at high risk of AMD. The overall findings from the AREDS2 and other studies suggest that L/Z is likely to be more appropriate and safer than beta-carotene in the original AREDS-type supplements [39,40]. The findings also point to the potential beneficial effects sufficient dietary intake of L/Z can have on AMD progression. Table 3 provides a summary of the findings from the AREDS2 study.

Evidence also suggests greater L/Z intake may particularly protect against AMD in persons with high genetic risk based on two major AMD genes [41]. Using data from the Blue Mountains Eye Study (BMES) and Rotterdam Study (RS), among participants with high genetic risk, the highest tertile of dietary intake of L/Z was associated with more than 20% reduced risk of early AMD (*p* = 0.0009). No similar association was, however, found for those with low genetic risk [41]. These findings are similar to those documented in Ho et al. [36] which is summarised as part of Table 2.

Randomised controlled trials with L/Z supplements have been conducted evaluating how L/Z supplementation affects visual performance in individuals with established AMD [42,43,44,45,46,47,48,49]. A meta-analysis of these seven studies was conducted and published in 2015 [6]. Results of this meta-analysis suggest that L/Z supplementation is associated with significant improvements in visual acuity and contrast sensitivity in a dose-response manner [6]. Furthermore, a linear association was indicated between these improvements and macular pigment optical density (MPOD) increase [6].

MPOD, a measurement of the attenuation of blue light by the macular pigment, is linearly related to the amount of L/Z in the macula. Precise roles and mechanisms are not fully understood; however, it is thought that MPOD levels, particularly in the central retina, may impact AMD [50].

The majority of studies have shown an age-dependent decrease in MPOD levels and a lack of MPOD in AMD compared to healthy controls [51]. A 2016 meta-analysis of 20 randomised controlled trials found that lutein, zeaxanthin and meso-zeaxanthin supplementation improves MPOD in both healthy subjects and in AMD patients in a dose-response manner [52]. These improvements were also positively associated with increases in serum L/Z levels.

Further research supports the consumption of dietary sources of L/Z to improve MPOD, particularly for individuals with low MPOD at baseline. An example of this is a study in healthy adults which compared the effect of L/Z-rich foods and supplements on MPOD as well as serological markers of endothelial activation, inflammation and oxidation. The eight- week intervention [53] which used spinach powder as the L/Z-rich food found improvements in MPL in the highest serum responders and in those with initially low MPL, supporting observational evidence to date. Another study showed the consumption of six eggs per week significantly increased the concentration of macular carotenoids (as measured by MPOD) as well as serum zeaxanthin levels; however, serum lutein levels remained unchanged [54]. Further evidence is warranted to better ascertain how different L/Z-containing foods may impact MPOD and which individuals would benefit from L/Z intake given there is evidence which suggests that the structural components responsible for the storage and availability of L/Z in the eye are genetically determined [50].

## 5. Recommended Intake Values for Lutein and Zeaxanthin

There are currently no official recommended dietary intake levels for L/Z. However, based on a paper published in 1994 [31], an intake of 6 mg of L/Z per day for men and women has been suggested as a dietary target to reduce the risk of age-related macular degeneration [55]. However, evidence from both the AREDS2 study and the BMES suggest that levels of intake of L/Z considerably less than 6 mg are associated with a decreased likelihood of AMD indicating lower levels of intake may be sufficient to provide some protection from progression of the disease, although further intervention studies to ascertain an appropriate target level, particularly for individuals at high risk of AMD, is needed. 

Data on population L/Z intake is limited, but current evidence suggests the intake of carotenoids varies in different populations as well as by season [56]. For example, in Europe, the average daily intake of major carotenoids (including retinol, alpha-tocopherol, beta-carotene, alpha-carotene, beta-cryptoxanthin, lutein, zeaxanthin, and lycopene) ranges from 3.5 mg/day in the Spanish population [57] to 5.33 mg/day in the German population [58]. Fewer studies have specifically measured L/Z consumption in populations, although in an American study of older adults, daily consumption of L/Z was 2.7 mg for men and 3.09 mg for women [59]. In another study, it was estimated that American adults consume approximately 1−2 mg of lutein per day [60]. In an Australian study of older adults living in the Blue Mountains, it was found that the average intake of L/Z was 0.9 mg and that women had slightly higher intakes than men [61]. The main contributors to L/Z intakes were broccoli, green beans and oranges. No other Australian studies have assessed the intake of these antioxidants in other population sub-groups; however, based on these results, current intakes in older adults are lower than ideal. 

In countries including Australia and the US, the national intakes of lutein may be declining. According to US National Health Interview Studies, there was a decrease in lutein consumption amongst different categories of people between 1987 and 1992 due to decreased intakes of green leafy vegetables [62]. In Australia, the National Nutrition Survey from 1983, 1985 and 1995 identified that between 1983 and 1995, the mean daily intake of fruit decreased by between 30 g and 50 g for men and women, respectively, and that the mean daily intake of vegetables decreased by 11 g for any population assessed [63]. Results from the latest Australian Health Survey indicated that only 7% of Australian adults are consuming the recommended five or more servings of vegetables per day [64]. Overall, the available data on current intakes suggests they are considerably below the current suggested intake of 6 mg per day. 

## 6. Dietary Sources of Lutein and Zeaxanthin

One of the difficulties in understanding and deciphering the exact role of dietary L/Z in AMD prevention and/or progression is the challenges inherent in analyzing and quantifying the L/Z content of foods. Until recently, L/Z were measured together because analytical procedures did not permit separate quantification of these carotenoids in foods. Because of this, composition tables commonly report the L/Z content of foods as one figure; however, testing of the individual carotenoids has been conducted [65] to ascertain the differing amounts of the individual carotenoids in foods. Given that L/Z accumulate in different regions of the eye and contribute to different functions, it may be important for both carotenoids to be consumed as part of a dietary pattern [65]. Accurate assessment of L/Z individually is crucial in evaluating their relative roles in eye health [65]. As shown in Table 4, the majority of vegetable sources of L/Z contain only lutein, whereas corn and eggs contain both lutein and zeaxanthin.

It is also now suggested that meso-zeaxanthin may not be solely derived from retinal lutein and that it may in fact be present in small amounts in the food supply [12]. For example, meso-zeaxanthin is reported to be commonly used in hen feed in Mexico to enhance the colouration of the egg yolk [12]. These findings suggest that it may be important for all three macular pigment carotenoids, (lutein, zeaxanthin and meso-zeaxanthin) to be quantified in food. Table 5 shows the lutein and zeaxanthin contents of a number of common foods. 

From a whole diet perspective, the availability of carotenoids from different types of foods i.e., vegetables, nuts and eggs, allows for a more varied and potentially more easily maintainable dietary pattern delivering higher amounts of L/Z. One concern with dietary interventions to increase levels of macular pigment has been the perception that the quantities of vegetables (in particular green leafy vegetables such as spinach) required to increase L/Z levels adequately are unrealistic; however, a careful, targeted selection of foods containing L/Z can still provide variety.

Dietary models demonstrating this variety as well as what dietary intakes of L/Z at various levels look like are outlined in Table 6 and Table 7. Two L/Z intake target levels were chosen given that: (1) work has previously suggested a dietary target of 5−6 mg L/Z [31]; and (2) 10 mg represents an upper level of what is achievable through dietary intake and is close to the L/Z level used in the AREDS supplement study [38]. While the dietary models are based on these higher levels of intake, evidence from cohort studies suggests lower dietary intakes of approximately 2.5 mg may be high enough to offer some protection against AMD development [21,38].

## 7. Absorption and Bioavailability of Dietary Carotenoids

While the total amount and distribution of L/Z in the food supply is important, it is also necessary to take into account factors that affect the absorption and bioavailability of these carotenoids. For example, the consumption of fat (in the form of a salad dressing, cooking oil such as extra virgin olive oil, or whole eggs) at the same meal as carotenoid intake (e.g., a raw vegetable salad or cooked vegetables) has been shown to effectively increase the absorption of some carotenoids [67,68,69,70,71].

Bioavailability of carotenoids may be decreased due to competition for absorption between carotenoids when consumed within the same meal [72]. Furthermore, dietary fibre from plant sources, such as pectin and guar gum, has been shown to reduce carotenoid absorption [73] and the localisation of carotenoids within the chloroplasts and chromoplasts of plants may decrease bioavailability [74]. The available research investigating the impact of cooking on carotenoids in plant sources suggests that although the heat decreases the carotenoid content, it may nonetheless enhance the bioavailability of the carotenoids compared with uncooked sources [69]. 

A particular example of a whole food where the bioavailability of L/Z may be higher than that of other sources is eggs. As seen in Table 5, the amount of L/Z in eggs is considerably lower than most L/Z-containing vegetables; however, research suggests the bioavailability of these compounds from eggs is higher than from vegetable sources, most likely due to the fat content [75]. The consumption of one egg per day over five weeks has been shown to increase serum lutein levels by 26% and zeaxanthin levels by 38% [76]. Other research has found the daily intake of three eggs for 12 weeks increased serum lutein and zeaxanthin by 21% and 48%, respectively, in 20 adults with the metabolic syndrome by increasing plasma HDL cholesterol [77].

A number of studies have also assessed the bioavailability of L/Z from eggs including eggs enriched with higher amounts of L/Z compared to standard eggs. One study showed the consumption of lutein- or zeaxanthin-enriched eggs or a lutein-enriched egg-yolk-based buttermilk beverage increased serum lutein levels significantly by 76% and 77%, respectively (*p* < 0.001) [78]. A high increase in the serum zeaxanthin concentration was also observed in individuals receiving zeaxanthin-enriched eggs: 430% (*p* < 0.001). These serum increases are comparable with daily use of 5 mg supplements [78]. 

## 8. Conclusions

Current evidence suggests that higher dietary intakes of lutein and zeaxanthin are likely to play an important role in protecting against age-related macular degeneration (AMD). Improvements in understanding and quantifying lutein and zeaxanthin (L/Z) as well as meso-zeaxanthin are needed to determine a recommended target for these macular carotenoids, and this will also help in better understanding their distinct roles in eye health. A diet high in a variety of foods is important for achieving adequate dietary levels of L/Z (as well as other nutrients). Moreover, such a diet should include plenty of leafy green vegetables, in keeping with dietary guidelines. There is also value in including a range of other foods to increase variety and improve the bioavailability of L/Z, such as eggs and selected nuts as part of a healthy dietary pattern. Intervention trials investigating the effectiveness of specific dietary patterns aimed at increasing macular pigment and preventing or delaying the progression of AMD are warranted. In the meantime, prudent advice to increase consumption of lutein- and zeaxanthin-containing foods in the diet of those people at high risk of AMD or who already have AMD should be encouraged.

## Figures and Tables

**Table 1:** Clinical classification scale for age-related macular degeneration [16].

Classification	Symptoms
No signs of AMD	No visible drusen or pigmentary abnormalities
No clinically significant increased risk of late AMD	Small drusen (<63 µm)
Early AMD	Medium drusen (≥63–<125 µm) without pigmentary abnormalities thought to be related to AMD
Intermediate AMD	Large drusen or pigmentary abnormalities associated with at least medium drusen
Late AMD	Lesions associated with neovascular AMD or geographic atrophy

**Table 2:** Summary of epidemiological evidence of dietary lutein/zeaxanthin intake and age-related macular degeneration.

**Cohort Studies**					
**Study**	**Length of follow-up Study participant details**	**Dietary Lutein and Zeaxanthin Intake**	**Dietary Assessment Method**	**Association between lutein and zeaxanthin consumption and Early or Intermediate Age-Related Macular Disease**	**Association between lutein and zeaxanthin consumption and Late Age-Related Macular Disease**
Van den Langenberg 1998 Beaver Dam Eye Study [29]	5-year follow-up of 1709 US men and women aged 43–84 years	Median intake (µg per 1000 kcal): Quintile 1: 294 µg Quintile 5: 1006 µg	FFQ (100 item)	No significant association between L/Z intake and early AMD.	Not reported
Van Leeuwen et al. 2005 The Rotterdam Study [28]	8-year follow-up of 4170 Dutch men and women aged ≥ 55 years	Mean intake mg/day: Quartile 1:1.4 mg Quartile 4:3.6 mg	FFQ (170 item)	No significant association between L/Z intake and incident AMD.	Not reported
Moeller et al. 2006 Carotenoids in Age-Related Eye Disease Study [33]	7-year follow-up of 1787 US women aged 50−79 years	Low (<28th percentile): 792 ± 169 µg/day vs. High (>78th percentile): 2868 ± 919 µg/day Median intake (µg per 1000 kcal): Low: 618 µg High: 1438 µg	FFQ (122 item)	No association between dietary L/Z and prevalence of intermediate AMD in high vs. low intake. Significant, protective association (OR: 0.57; 95% CI 0.34−0.95) in women <75 years with stable L/Z intake and no history of CVD, diabetes, hypertension and/or AMD.	No significant association between dietary L/Z levels and late AMD in overall sample ORs in protective direction in younger women.
Tan et al. 2008 Blue Mountains Eye Study [34]	10-year follow-up of 2454 Australian men and women aged 45−93 years	Median intake µg/day: 743 µg (SD: 482 µg) Top tertile: ≥ 942 µg/day	FFQ (145 item)	Those with above-median intakes had a reduced risk of indistinct soft or reticular drusen (RR: 0.66; 95% CI, 0.48−0.92).	Top tertile of L/Z intake had a reduced risk of incident neovascular AMD (RR: 0.35, 95% CI 0.13–0.92).
Cho et al. 200 The Health Professionals Follow-Up Study [35]	16-year follow-up of 41,564 US male health professionals aged 50−79 years	Quintile 1: 1209 ± 317 µg/day Quintile 5: 6879 ± 315 µg/day	FFQ (130 item)	No overall association between L/Z intake and early AMD risk.	Top vs. bottom quintiles of L/Z intake and neovascular AMD pooled multivariate RR 0.78 (95% CI: 0.57, 1.06)
Cho et al. 2008 The Nurses’ Health Study [35]	18-year follow-up of 71,494 US female health professionals aged 50−79 years	Quintile 1: 1097 ± 279 µg/day Quintile 5: 5852 ± 2797 µg/day	FFQ (130 item)	No overall association between L/Z intake and early AMD risk.	Top vs. bottom quintiles of L/Z intake and neovascular AMD pooled multivariate RR 0.78 (95% CI: 0.57, 1.06)
Ho et al. 2011 The Rotterdam Study [36]	8.6-year follow-up 2167 individuals (≥ 55 years) from the Rotterdam study at risk of AMD	Tertile 1: 1.45−1.50 mg/day Tertile 3: 3.29−3.39 mg/day	FFQ (170 item)	High dietary intake of L/Z reduced the risk of early AMD in those at high genetic risk.	Not reported
**Case-Control Studies**						
Seddon et al. 1994 The Eye Disease Case-Control Study [31]	Case-control study of 356 subjects aged 55 to 80 years with advanced-stage AMD matched with 520 control subjects	Median intake (IU): Quintile 1: 560.8 Quintile 5: 5757	FFQ (60 item)	Not reported	Highest vs. lowest dietary L/Z was associated with a reduced risk for advanced AMD (OR: 0.43; 95% CI 0.2−0.7).
Snellen et al. 2002 [37]	Case-control study of 72 case and 66 control subjects	Levels not reported.	Dietary habits interview	Not reported	The odds ratio for neovascular AMD with low L/Z vs. high L/Z was OR: 2.4 (95% CI, 1.1−5.1).
SanGiovanni et al. 2007 The Age-Related Eye Disease Study (AREDS) Report 22 [21]	Case-control study of 4519 AREDS participants aged 60 to 80 years at baseline	Median intake (µg per 1000 kcal): Quintile 1: 521 µg Quintile 5: 2095 µg	FFQ (90 item)	Highest quintile vs. lowest quintile dietary L/Z was inversely associated with large or extensive intermediate drusen (OR: 0.73; 95% CI 0.56−0.96).	Highest quintile vs. lowest quintile dietary intake L/Z was inversely associated with neovascular AMD (OR: 0.65; 95% CI 0.45−0.93) and geographic atrophy (OR: 0.45; 95% CI 0.24−0.86).

Abbreviations: FFQ, food frequency questionnaire; OR, odds ratio; CVD, cardiovascular disease; SD, standard deviation.

**Table 3:** Summary of randomised controlled trials regarding lutein and zeaxanthin intake and AMD.

Study	Length of Follow-Up	Study Participants	Lutein and Zeaxanthin Intake	Lutein and Zeaxanthin Intake and Risk of Early AMD	Lutein and Zeaxanthin Intake and Risk of Late AMD
AREDS II [38]	5 years	4203 US adults aged 50−85 years at risk of progression to late AMD	Lutein (10 mg) + zeaxanthin (2 mg) supplements plus baseline median intake of dietary L/Z of 2590 µg/day	Not reported	Lutein and zeaxanthin supplementation (as an addition to the original AREDS supplement) lowered the progression to late AMD but only in individuals with low dietary lutein and zeaxanthin. A reduction in risk for progression to late AMD was seen for individuals in quintile 1 who had a median intake of 696 µg L/Z per 1000 cal per day compared to no L/Z (HR: 0.74; 95% CI, 0.59−0.94). For those with a median intake of 1134 µg per 1000 cal per day—HR: 0.94 (95% CI: 0.74−1.21).

**Table 4:** Individual lutein and zeaxanthin values of common foods [65].

Food	Lutein Trans (µg per 100 g)	Zeaxanthin Trans (µg per 100 g)	L/Z Ratio ^1^
Asparagus, cooked	991	0	-
Spinach, raw	6603	0	-
Spinach, cooked	12,640	0	-
Kale, cooked	8884	0	-
Green beans, cooked	306	0	-
Orange pepper, raw	208	1665	0.1
Lettuce, romaine, raw	3824	0	-
Broccoli, cooked	772	0	-
Parsley, raw	4326	0	-
Corn, cooked	202	202	1.0
Pistachio nuts, raw	1405	0	-
Egg whole, cooked	237	216	1.1
Egg yolk, cooked	645	587	1.1
Egg whole, raw	288	279	1.0
Egg yolk, raw	787	762	1.0

^1^ Ratio of all-trans lutein to all-trans zeaxanthin.

**Table 5:** Lutein and zeaxanthin content of common foods [66].

Food	Lutein and Zeaxanthin (µg/100 g)
Kale, cooked	18,246
Spinach, raw	12,197
Spinach, cooked	11,308
Parsley	5562
Peas, green (boiled)	2593
Lettuce (romaine or cos)	2313
Squash (boiled)	2249
Edamame beans	1619
Brussels sprouts (boiled)	1541
Pistachio nuts, raw	1404
Egg yolk, raw	1094
Broccoli (cooked)	1079
Pumpkin (cooked)	1014
Asparagus, cooked	771
Frozen corn (boiled from frozen)	684
Frozen green beans (cooked)	564
Egg whole, raw	504
Egg whole, cooked (hard-boiled)	353
Avocado (all commercial)	270
Orange (all commercial)	129
Tomato (red, ripe, cooked)	94

**Table 6:** Example meal plan with calculated L/Z content >5 mg.

Meal	Lutein/Zeaxanthin Content
**Breakfast:** Porridge with nuts: Porridge (rolled oats, milk, water) topped with 30 g **pistachio nuts** and 1/2 tsp honey + one medium **orange**	589 µg
**Lunch:** Curried egg sandwich: one boiled **egg** mixed with mayonnaise and curry powder, two slices wholegrain bread, salad vegetables including **baby spinach**	4026 µg
**Dinner:** Salmon, rice and vegetables: Oven-baked salmon fillet, **corn on the cob**, steamed vegetables including **asparagus** and **broccoli**	1729 µg
**Snacks**: One cup **blueberries** + cheese and crackers	118 µg
**Total daily L/Z intake**	6462 µg

**Table 7:** Example meal plan with calculated L/Z content >10 mg.

Meal	Lutein/Zeaxanthin Content
**Breakfast:** Scrambled eggs on toast: Two poached **eggs** on wholegrain toast served with 1/2 cup wilted **kale** and grilled **tomato** + one glass milk	9584 µg
**Lunch:** Cheese and salad wrap: one wholegrain wrap with hummus, salad vegetables (**cos lettuce**, cucumber, carrot), 1/3 cup grated cheese and 1/4 **avocado** + one medium **orange**	942 µg
**Dinner**: Lamb chops and vegetables: Trim lamb chops with roast **pumpkin** and mixed vegetables (cooked in extra virgin olive oil)	760 µg
**Snacks**: one cup fresh fruit salad + one tub natural yoghurt	Nil
**Total daily L/Z intake**	**11,286 µg**

## Supplementary Materials

The following are available online at http://www.mdpi.com/2072-6643/9/2/120/s1, Figure S1: Intermediate AMD; Figure S2: Neovascular late AMD.
