# Supplementary material for: Lutein and Zeaxanthin—Food Sources, Bioavailability and Dietary Variety in Age-Related Macular Degeneration Protection
Source: Nutrients. 2017 Feb 9;9(2):120. doi: 10.3390/nu9020120 (PMC5331551; doi:10.3390/nu9020120)
Supplement: Supplementary file 1 [file nutrients-09-00120-s001.docx]

Supplementary Materials: Lutein and Zeaxanthin—Food Sources, Bioavailability and Dietary Variety in Age-Related Macular Degeneration Protection

Bronwyn Eisenhauer, Sharon Natoli, Gerald Liew and Victoria M. Flood


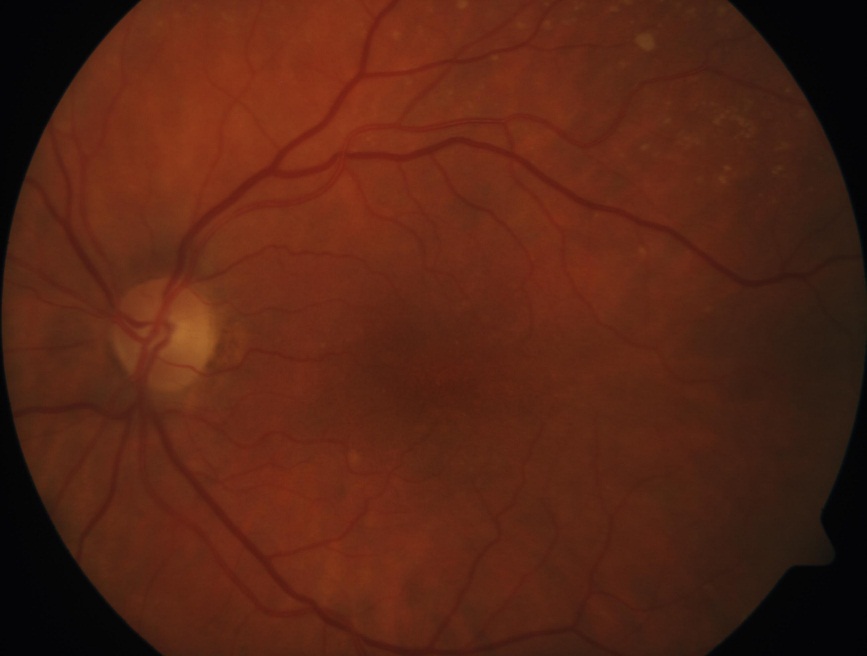


**Figure S1.** Intermediate AMD.


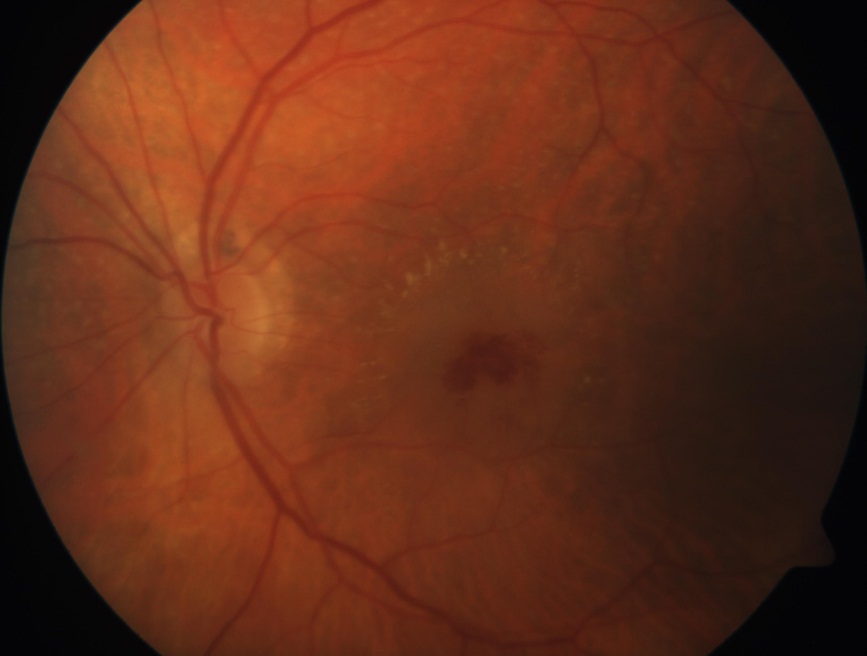


**Figure S2.** Neovascular late AMD.
